# A particular epidemiological profile: disparities in access to contraceptive methods in Brazil during the SARS-CoV-2 (COVID-19) pandemic

**DOI:** 10.1038/s41598-024-65946-w

**Published:** 2024-07-01

**Authors:** Ana C. Marcelino, Paula da Cunha Pereira, Charles M.’Poca Charles, Luis Bahamondes

**Affiliations:** Department of Obstetrics and Gynecology, University of Campinas Faculty of Medical Sciences, Caixa Postal 6181, Campinas, SP 13084-971 Brazil

**Keywords:** Epidemiology, Health policy

## Abstract

Our objective was to study disparities in access to contraception during the COVID-19 pandemic. We performed a cross-sectional study at the University of Campinas, Brazil using a Google questionnaire applied from December 2021 until February 2022, disseminated via snowball technique. The survey asked about sociodemographic characteristics and contraceptive use, as well as the demand for new methods and difficulties in continuing to use contraceptives during the COVID-19 pandemic. We analyzed 1018 completed questionnaires; in total, 742 (72.9%) were women aged between 20 and 39 years, 746 (73.3%) were White and 602 (59.2%) used contraceptives. During the COVID-19 pandemic, about 23% of respondents changed their method and approximately 20% of respondents looked for new methods. Among the latter, 31.3% reported some difficulty with obtaining guidance on new methods while only 5.3% of the respondents reported some difficulty with continuing their contraceptive. The main difficulty in both cases was the difficulty with getting a healthcare provider appointment. Our results point to a particular epidemiological population, of younger black and biracial women, with lower education and lower income, which suffered health disparities during the COVID-19 pandemic and found difficulties with using contraceptives and accessing family planning services.

## Introduction

The first case of SARS-CoV-2 (COVID-19) in Brazil was confirmed on February 26, 2020; since then, more than 38,000,000 cases of the disease were reported, with a total of 708,373 deaths^1^. In the midst of government measures that have demonstrated a lack of coordination in preventive measures^2^, especially in priority groups^3^, Brazil has gone through three epidemiological waves of COVID-19^4^, having prematurely declared the end of the Public Health Emergency of National Concern by COVID-19 on April 22, 2022^5^. However, the World Health Organization only declared the end of the Public Health Emergency of International Concern regarding COVID-19 on May 5, 2023^6^.

For more than three decades, Brazil has had the National Health System (*Sistema Unificado de Saúde*, SUS), which provides universal, equitable and integral health services for citizens; its resilience was tested during the pandemic in many ways. Family planning activities were questioned regarding their essential nature and there were reports of interruptions in these services because they were considered non-essential by the Federal and State governments. At the time, experts were already warning about the possible effects of this interruption and the need for family planning services to remain open^7–9^.

These concerns have been confirmed with reports of a lack of access to contraceptives, mainly long-acting reversible contraceptives (LARCs), and services, as well as an increase in unplanned pregnancies worldwide during the pandemic, alongside other impacts on sexual and reproductive health (SRH)^10,11^. Particularly in cases of abortion, the relationship between the disruption in family planning services and access to abortion services and its complications is clear^12^; the impact of the COVID-19 pandemic on pregnancy was also evident, with an increase in negative pregnancy outcomes such as prematurity cases^13^, an increase in maternal and fetal death and an increase in the cesarean delivery rate, mainly in low socioeconomic groups, with less schooling and black and biracial ethnicity women^14–16^.

More and more evidence is being shared on health disparities around the world^17,18^, which have been made clear by the pandemic^14,19–22^. A particular epidemiological profile is evident, especially in terms of education, income and ethnicity^23^. Given the impact of family planning to reduce unplanned pregnancies and their health consequences, studying disparities in access to contraception is important because of its ability to mitigate suffering and social inequality.

## Methods

We conducted a cross-sectional study using an online questionnaire developed by the family planning outpatient team of the Department of Obstetrics and Gynecology, University of Campinas Faculty of Medical Sciences, Brazil. The university of Campinas ethics committee approved the research protocol. Using Google Form (Google LLC, Mountain View, CA, USA), we designed an online, self-administered questionnaire, whose answers were not mandatory, applied from December 3, 2021, until February 8, 2022. A pilot version of the questionnaire was tested on 15 women, doctors and nurses at the family planning clinic to ensure its clarity and the time it would take to complete.

We invited self-identified women without any exclusion criteria via social media platforms (WhatsApp and Facebook, Instagram) to answer the questionnaire. We used the snowball technique to disseminate the study by asking women who were working at the family planning clinic, as well as using the family planning clinic´s social media profiles to disseminate the study, and we also asked the respondents to send the questionnaire to their women contacts on social media.

When accessing the online questionnaire and reading an introduction explaining its contents, an Informed Consent Form was provided; it was only possible to continue with the questionnaire after confirming adulthood and consent. After each participant completed the questionnaire, the form was directed automatically to an Excel sheet generated by Google. When analyzing the answers, we excluded post-menopausal women, pregnant women and hysterectomized women. The entire study was carried out followed the relevant Brazilian and international ethical guidelines and regulations.

The survey asked about sociodemographic characteristics, history of COVID-19 infection, disease or vaccination, current contraceptive use, the demand for new methods and difficulties with continuing to use contraceptives during the COVID-19 pandemic. They were also asked about their menstrual cycle during the pandemic, as well as infection and vaccination against COVID-19; this information was published elsewhere^24^.

We asked about the use of current contraceptives and whether it was difficult to keep using the method, as well as if it was difficult to get guidance on new methods, grading the answers from very easy to very difficult. If the answer was difficult or very difficult, they were asked why and could choose between the options: difficult to find the method, difficult to buy the method, I couldn't get a provider's appointment for advice, I can't renew my prescription, or other. The respondents were also asked if they had changed contraceptives and, if so, to which method.

### Analysis

We first described our sample and the reported use of contraceptives. We then described any difficulties with continuing to use the method and, when there was difficulty, we described why. We described whether there had been any change in method and if so to which method. We also described whether there had been a change in method, whether there had been any difficulty with finding guidance on methods and if there had been difficulty, the main reasons. Finally, we explored potential relationship between the difficulties with maintaining contraceptive use and the demand for new methods with socio-economic variables, including age, ethnicity, years of schooling, occupation, family income, contraceptive use (yes or no) and type.

We performed descriptive analysis with frequency tables for categorical variables. The Chi-square test or Fisher's exact test were used to verify associations or compare proportions, when necessary. The level of significance adopted for the statistical tests was 5%. We used SAS version 9.4 (SAS Institute Inc, 2002–2008, Cary, NC, USA) software for all analyses.

### Ethical approval

The university of Campinas ethics committee approved the research protocol. When accessing the online questionnaire and reading an introduction explaining its contents, an Informed Consent Form was provided; it was only possible to continue with the questionnaire after confirming adulthood and consent. The entire study was carried out followed the relevant Brazilian and international ethical guidelines and regulations.

## Results

We received 1060 questionnaires, the answers for which were not compulsory; the data for missing answers are described in the tables. In total, 11 questionnaires were excluded because they were more than 30% incomplete, 17 were excluded because the respondents were pregnant and 14 were excluded because they were hysterectomized women, leaving a total of 1018 responses to be analyzed. Of these, 742 (72.9%) women were aged between 20 and 39 years, 746 were White (73.3%) and 602 (59.2%) were using contraceptives at the time of the questionnaire (Table 1). Only 5.3% of the respondents reported some difficulty with continuing contraceptive use, with most of the answers being equal difficulty or easy (Table 2). However, among those who found it difficult or very difficult, the main reason was not being able to get a healthcare provider's appointment or difficulties with paying for the method (Table 2).

**Table 1:** Sociodemographic, clinical and gynecologic characteristics of the questionnaire respondents, Brazil, (n = 1018).

Characteristics	n	%
*Age, year (n* = *1018)*		
18–19	35	3.4
20–29	410	40.3
30–39	332	32.6
≥ 40	241	23.6
*Ethnicity (n* = *1018)*		
White	746	73.3
Black and biracial	249	24.4
Others	23	2.3
*Cohabitation status (n* = *1017)*		
Without a partner	523	51.4
Years of schooling* (n = *1018)*		
less than 12	35	3.5
12	171	16.8
13 or more	812	79.8
*Occupation (n* = *1016)*		
Student	217	21.4
Unemployed	65	6.4
Self-employed professional	292	28.7
Registered professional	324	31.9
Other	118	11.6
Family income (in Brazilian Reals)** (n = *1016)*		
≤ 3300	254	24.9
3301–5500	182	17.9
> 5500	580	57.1
*BMI (n* = *1013)*		
≤ 17.9	21	2.1
18–-24.9	570	56.3
25–29.9	234	23.1
≥ 30	188	18.5
*Current contraceptive use (n* = *1017)*		
Yes	602	59.2
*Current contraceptive method (n* = *1011)*		
Hormonal-IUD	146	14.4
Copper-IUD	89	8.8
Subdermal implant	51	5.0
Injectable/pill/ring/patch	242	24.0
None/behavioral methods/barrier	449	44.4
Permanent	34	3,4
*Parity (n* = *1018)*		
0	595	58.4
1–2	318	31.3
≥ 3	105	10.3
*COVID-19 infection (n* = *1018)*		
Yes	422	41.5
*COVID-19 vaccination (n* = *1013)*		
Yes	1000	98.7

BMI, body mass index (kg/m^2^); IUD, intrauterine device.

*In Brazil, basic education consists of 12 full years of study.

**In Brazil, the minimum recommended worker's income is defined as the “minimum wage” which is worth R$1320, equivalent to US$256.85.

**Table 2:** Difficulty in using contraceptives and main barriers to access during the COVID-19 pandemic for the questionnaire respondents, Brazil, (n = 1018).

	n	%
*Difficulty to keep up using this contraceptive method during the pandemic (n* = *1011)*		
Very difficult	11	1.1
Difficult	42	4.2
Same	372	36.8
Easy	103	10.2
Very easy	205	20.3
Not using method	278	27.5
*Reason for difficulty (if difficult or very difficult) (n* = *52)*		
Difficult to find the method	7	13.5
Difficult to buy the method	13	25.0
I couldn't get a doctor's appointment	17	32.7
I couldn't get my prescription renewed	4	7.7
I didn't adapt to the method	11	21.2
*Changed contraceptive during the pandemic (n* = *985)*		
Yes	226	22.9
No	759	77.1
*If yes, for which contraceptive (n* = *224)*		
I stopped using any method	47	21.0
Behavioral methods/barrier	27	12.1
Hormonal-IUD	54	24.1
Copper-IUD	33	14.7
Subdermal implant	19	8.5
Injectable/pill/ring/patch	41	18.3
Permanent	3	1.3

About 23% of respondents changed their method during the COVID-19 pandemic (Table 2) and approximately 20% of respondents looked for new methods during the COVID-19 pandemic (Table 3). Among the latter, 31.3% reported some difficulties with obtaining guidance on new methods during the pandemic (Table 3). The main issue in this regard was the difficulty with getting a medical consultation (56.5%). The analysis of changing methods and seeking new methods during the pandemic according to current contraceptive use (yes/no) showed that most women who did not use methods did not seek them (87.8%) or did not change methods (85.7%), while 28.5% of women who already used methods changed their method and 25.8% sought new ones (*p* < 0.0001 in both situations). The analysis between difficulty obtaining information about methods during the pandemic and the use of current contraceptives showed no significance.

**Table 3:** Demand for new methods during the during the COVID-19 pandemic and main barriers to access of the questionnaire respondents, Brazil, (n = 1018).

	n	%
*Did you look for new contraceptives during the pandemic? (n* = *995)*		
Yes	204	20.5
No	791	79.5
*Difficulty obtaining guidance on new contraceptive methods during the pandemic (n* = *198)*		
Very difficult	18	9.1
Difficult	44	22.2
Same	49	24.8
Easy	52	26.3
Very easy	35	17.7
*Reason for difficulty (if difficult or very difficult) (n* = *62)*		
Difficult to find the method	15	24.2
Difficult to buy the method	8	12.9
I couldn't get a doctor's appointment	35	56.5
I couldn't get a prescription	2	23.2
Other	2	23.2

Analysis of the difficulty with continuing to use their existing method during the COVID-19 pandemic and variables shows a relationship with ethnicity, years of schooling, occupation and family income, with greater difficulty reported among black and biracial women, those with fewer years of schooling, unemployed women and those with lower incomes (Table 4).

**Table 4:** Difficulty in maintaining contraceptive use, stratified by age, ethnicity, years of schooling, occupation and family income, Brazil, (n = 596).

	Difficulty						
	Very easy and easy (n = 250)		Same (n = 298)		Difficult and very difficult (= 48)		*p* value*
	n	%	n	%	n	%	
*Age, years*							
18–19	9	3.6	12	4.0	5	10.4	0.3760*
20–29	122	48.8	155	52.0	24	50.0	
30–39	75	30.0	92	30.9	11	22.9	
> 40	44	17.6	39	13.0	8	16.7	
*Ethnicity*							
White	193	77.2	218	73.2	20	41.7	< 0.0001**
Black and biracial	52	20.8	74	24.9	27	56.3	
Others	5	2.0	6	2.0	1	2.1	
Years of schooling***							
Less than 12	08	3.2	14	4.7	3	6,3	< 0.0001*
12	50	20.0	42	14.1	21	43.8	
13 or more	192	76.8	242	81.2	24	50.0	
*Occupation*							
Student	61	24.4	77	25.8	12	25.0	0.0007**
Unemployed	10	4.0	17	5.7	10	20.8	
Self-employed professional	66	26.4	94	31.5	11	22.9	
Registered professional	89	35.6	86	28.9	15	31.3	
Other	24	9.6	24	8.1	0	0	
Family income****							
≤ 3300	53	21.2	72	24.2	31	64.6	< 0.0001*
3301–5500	51	20.4	51	17.1	2	4.2	
> 5500	145	58.2	174	58.6	15	31.3	

*Based on Fisher's exact test/**based on Chi-square test.

***In Brazil, basic education consists of 12 full years of study.

****In Brazil, the minimum recommended worker's income is defined as the “minimum wage” which is worth R$1320, equivalent to US$256.85.

Finally, the analysis between the demand for new methods during the COVID-19 pandemic shows a relationship with age, years of schooling, occupation, family income and current contraceptive method, with greater demand reported up to the age of 39 years, in those with fewer years of schooling and among students, registered workers and low-income individuals (Table 5). Most of the women who changed their method opted to use copper or hormonal intrauterine devices (IUDs), while most of the women who did not change their contraceptives during the pandemic either did not use any method or used the pill (Table 5). There was no statistical significance for the relationship between the ease of finding new methods and the demographic characteristics analyzed.

**Table 5:** Demand for new contraceptive methods during the COVID-19 pandemic, stratified by age, ethnicity, years of schooling, occupation, family income, and contraceptive method of the respondents, Brazil, (n = 995).

	Demand for new methods				
	Yes (n = 204)		No (n = 791)		*p* value
	n	%	n	%	
*Age, Years*					
18–19	11	5.4	24	3.0	< 0.0001*
20–29	109	53.4	292	36.9	
30–39	64	32.4	263	33.2	
> 40	20	9.8	212	26.9	
*Ethnicity*					
White	140	68.6	587	74.2	0.2928**
Black and biracial	61	29.9	184	23.2	
Others	3	1.5	20	2.5	
Years of schooling***					
Less than 12	14	6.9	20	2.6	< 0.0001**
12	50	24.5	117	14.8	
13 or more	140	68.6	654	82.7	
*Occupation*					
Student	58	28.4	153	19.4	0.0009*
Unemployed	16	7.8	47	6.0	
Self-employed professional	40	19.6	246	31.2	
Registered professional	74	36.3	247	31.3	
Other	16	7.8	96	12.2	
Family income****					
≤ 3300	71	35.0	178	22.5	0.0002*
3301–5500	32	15.8	140	18.3	
> 5500	100	49.3	468	59.2	
*Contraceptive method in use*					
Hormonal-IUD	46	22.5	100	12.7	< 0.0001 *
Copper-IUD	36	17.6	53	6.7	
Subdermal implant	11	5.4	40	5.1	
Injectable/pill/ring/patch	50	24.4	192	24.4	
None/behavioral methods/barrier	59	28.9	373	47.4	
Permanent	2	1.0	29	3.7	

*Based on Chi-square test/**based on Fisher's exact test.

***In Brazil, basic education consists of 12 full years of study.

****In Brazil, the minimum recommended worker's income is defined as the “minimum wage” which is worth R$1320, equivalent to US$256.85.

## Discussion

Family planning is crucial for the sustainable development of all individuals, as it enables people to achieve their reproductive goals while also reducing problems arising from unplanned pregnancies and abortions, as well as reducing the risk of maternal and infant mortality^25–27^. The impact of COVID-19 pandemic on family planning services and access to contraceptives has been highlighted by the United Nations, especially among young women and adolescents and in low- and middle-income countries, and thinking about SRH policies for gender equity during this period is even more fundamental^28,29^.

In our study, approximately 45% of the women did not use a method or used behavioral awareness methods or condoms, which is indicative of an unmet need for modern contraceptives that is also found in other countries^9^. A high proportion of women were not in a stable relationship, which may have influenced the decision to use short-acting contraceptive methods (SHAC) or not to use a method at all^9,30^.

The use of SHAC, such as the pill or injectables, which are available in pharmacies over the counter in Brazil, is around 24%. A study carried out in Brazil showed that sales of injectables and pills during the COVID-19 pandemic increased in the first months of the pandemic, coinciding with the closure of many family planning clinics, with a subsequent increase in the sale of hormonal-IUD and implants in the private sector, indicating an inequity against the poorest individuals^31^.

We also observed that almost 30% of participants were using LARC methods, which shows a higher use than the common methods^9^. Furthermore, almost 50% of the women who changed their contraceptive method chose and were able to obtain LARC, which may show the resilience of family planning services during the pandemic in Brazil and the importance of keeping these services open to the population using the national health service.

Regarding the demand for new methods, the majority did not change their method during the pandemic, with many who did not change their contraceptives being shown to not use a method or to be using the pill. This indicates that the pandemic did not lead to a change in the desire to become pregnant in the population studied. Despite this, approximately 20% sought new methods and changed their methods during the pandemic. Those who sought out new methods were mostly young women with 12 years or less of schooling, students and those with low incomes, which reinforces the gap in the unmet need for family planning^9,32^.

Despite representing approximately 5% of the sample, women who showed difficulties with maintaining contraceptive use during the pandemic have particular characteristics; the majority were black or biracial, with fewer years of schooling, were unemployed and had a low family income^3^. These inequities were also reported globally^33–35^.

Among the main reasons were difficulties with buying contraceptive methods, which may demonstrate the economic crisis faced during the pandemic by this part of the population^21,23^, especially according to recent national data showing that White people are paid 61.4% more than Black people (Black and biracial) per hour of work in Brazil^36^ and difficulties with getting a medical consultation; this may demonstrate the barriers to access imposed on the poorest, least educated and Black and biracial people^17,37,38^. In addition, around 30% found it difficult to obtain new information about contraceptive methods, with the main reason being that they were unable to find a healthcare provider appointment.

Efforts need to be increased in this specific epidemiological group, which consists of black or biracial women with fewer years of schooling, who are unemployed and have a low income, in order to maximize the effects of family planning, access to information and maintaining the use of methods. In particular, racism can be a reason for not obtaining or delaying appointments^17,37,39^. Racism in the use of health services is associated with more reports of negative experiences in the health service, delays or failure to obtain appointments and lack of adherence to the proposed treatment^37^. In Brazil, the chance of a Black or biracial person not being able to access health services is twice as high as for a White person^40^ and it has long been a fact that black and biracial women with lower levels of education and family income use less contraception and have more children^41^. In the case of family planning, its impact may be in the delay in consultations or barriers by professionals to prescribing or inserting LARC methods. Despite the universal access that Brazil's public health system provides, efforts still need to be focused on black or biracial women with fewer years of schooling, who are unemployed and have a low income, tackling barriers such as racism and social inequality, as well as relying on human rights principles^32^.

The main strength of our study is that the online questionnaire allowed us to collect data quickly, anonymously and with wide national coverage. In addition, the questionnaire was administered during the COVID-19 pandemic, reducing recall bias. The main limitation was that the sample was obtained by convenience, which may have contributed to the selection of predominantly White women, with a high level of education and higher incomes. However, this information is still relevant, demonstrating the epidemiological profile of the population that has access to the internet and has time to respond to surveys.

Future studies should focus on techniques or conditions that make it easier for underserved populations to improve the access to both information and family planning consultations, as well as reducing barriers when it comes to prescribing a method or starting a LARC methods. In view of the above results, and the evidence worldwide about disparities in sexual and reproductive health, it would be essential during public health crisis that policy makers considered that family planning services are essential, so that their activities are not delayed or suspended.

In conclusion, our results point to a particular epidemiological population, which has suffered health disparities during the COVID-19 pandemic, in the difficulty of using and accessing family planning services. It is essential, especially during pandemics when the course of health is uncertain, to mitigate such disparities and their negative health consequences, focusing on public health actions that guarantee such rights to younger, black and biracial women, with lower education and lower incomes.

## Data Availability

Due to the University of Campinas Ethical Committee restrictions the data are available only on request directly to the corresponding author Dr Luis Bahamondes.

## References

[1] Coronavírus Brasil. https://covid.saude.gov.br/

[2] Agostoni C, Ramacciotti K, Paiva CH, Cueto M (2023). COVID-19 in Latin America: Conflicts, resistances and inequalities. Hist. Ciênc. Saúde-Manguinhos.

[3] de Paula NM, Pereira W, Giordani RCF (2023). A COVID-19 em meio a uma “tempestade perfeita” no capitalismo neoliberal: Reflexões críticas sobre seus impactos no Brasil. Ciênc. Saúde Coletiva.

[4] Moura EC (2022). Covid-19: Evolução temporal e imunização nas três ondas epidemiológicas, Brasil, 2020–2022. Rev. Saúde Pública.

[5] Ministério da Saúde declara fim da Emergência em Saúde Pública de Importância Nacional pela Covid-19. *Ministério da Saúde*. https://www.gov.br/saude/pt-br/assuntos/noticias/2022/abril/ministerio-da-saude-declara-fim-da-emergencia-em-saude-publica-de-importancia-nacional-pela-covid-19

[6] OMS declara fim da Emergência de Saúde Pública de Importância Internacional referente à COVID-19–OPAS/OMS|Organização Pan-Americana da Saúde. https://www.paho.org/pt/noticias/5-5-2023-oms-declara-fim-da-emergencia-saude-publica-importancia-internacional-referente

[7] Bahamondes L, Makuch MY (2020). Family planning: An essential health activity in the pandemic of SARS-CoV-2. Eur. J. Contracept. Reprod. Health Care.

[8] Riley T, Sully E, Ahmed Z, Biddlecom A (2020). Estimates of the potential impact of the COVID-19 pandemic on sexual and reproductive health in low- and middle-income countries. Int. Perspect. Sex. Reprod. Health.

[9] *World Family Planning 2022: Meeting the Changing Needs for Family Planning: Contraceptive Use by Age and Method* (United Nations, 2022).

[10] Brunie A (2022). Women’s experiences with family planning under COVID-19: A cross-sectional, interactive voice response survey in Malawi, Nepal, Niger and Uganda. Glob. Health Sci. Pract..

[11] Karaahmet AY, Bilgiç FŞ (2022). COVID-19: the unmet need for family planning and its effects on sexuality: A cross-sectional study. Rev. Assoc. Médica Bras..

[12] Dantas PBF (2023). The impact of the COVID-19 pandemic on the care of women experiencing abortion in a university hospital in Brazil. RBGO Gynecol. Obstet..

[13] Charles CM (2023). Preterm births prevalence during the COVID-19 pandemic in Brazil: Results from the national database. Sci. Rep..

[14] Brioschi dos Santos AP (2023). The impact of COVID-19 on maternal death and fetal death, a cohort study in Brazil. PLoS ONE.

[15] Xavier MO (2023). The impact of the COVID-19 pandemic on trends in stillbirths, under-5 and maternal mortality in Brazil: Excess deaths and regional inequalities. J. Glob. Health.

[16] Guimarães RM (2023). Tracking excess of maternal deaths associated with COVID-19 in Brazil: A nationwide analysis. BMC Pregnancy Childbirth.

[17] Hall WJ (2015). Implicit racial/ethnic bias among health care professionals and its influence on health care outcomes: A systematic review. Am. J. Public Health.

[18] Petersen EE (2019). Racial/ethnic disparities in pregnancy-related deaths—United States, 2007–2016. Morb. Mortal. Wkly. Rep..

[19] Khanijahani A, Iezadi S, Gholipour K, Azami-Aghdash S, Naghibi D (2021). A systematic review of racial/ethnic and socioeconomic disparities in COVID-19. Int. J. Equity Health.

[20] Magesh S (2021). Disparities in COVID-19 outcomes by race, ethnicity and socioeconomic status: A systematic-review and meta-analysis. JAMA Netw. Open.

[21] da Silva SA (2021). A Pandemia de Covid-19 no Brasil: A pobreza e a vulnerabilidade social como determinantes sociais. Confins Rev. Fr. Brés. Géogr..

[22] Cardoso FS, Gomes DCK, da Silva AS (2023). Racial inequality in health care of adults hospitalized with COVID-19. Cad. Saude Publica.

[23] dos Santos HLPC (2020). Necropolitics and the impact of COVID-19 on the Black community in Brazil: A literature review and a document analysis. Ciênc. Saúde Coletiva.

[24] Marcelino AC (2024). Association between COVID-19 and vaccination on menstrual cycle. Int. J. Gynecol. Obstet..

[25] *The Millenium Development Goals Report 2012* (United Nations, 2012).

[26] Osotimehin B (2012). Family planning saves lives, yet investments falter. Lancet.

[27] Alkema L, Kantorova V, Menozzi C, Biddlecom A (2013). National, regional and global rates and trends in contraceptive prevalence and unmet need for family planning between 1990 and 2015: A systematic and comprehensive analysis. Lancet.

[28] Bailey MJ, Bart L, Lang VW (2022). The missing baby bust: The consequences of the COVID-19 pandemic for contraceptive use, pregnancy and childbirth among low-income women. Popul. Res. Policy Rev..

[29] Heidari S (2023). A gender-responsive pandemic accord is needed for a healthier, equitable future. Lancet.

[30] Borges ALV (2023). Changes in contraceptive use during the second COVID-19 lockdown in Brazil: A web-based survey. Contraception.

[31] Charles CM, Munezero A, Bahamondes LG, Pacagnella RC (2022). Comparison of contraceptive sales before and during the COVID-19 pandemic in Brazil. Eur. J. Contracept. Reprod. Health Care.

[32] Cottingham J, Germain A, Hunt P (2012). Use of human rights to meet the unmet need for family planning. Lancet.

[33] Diamond-Smith N (2021). COVID-19’s impact on contraception experiences: Exacerbation of structural inequities in women’s health. Contraception.

[34] Maier M, Samari G, Ostrowski J, Bencomo C, McGovern T (2021). ‘Scrambling to figure out what to do’: A mixed method analysis of COVID-19’s impact on sexual and reproductive health and rights in the United States. BMJ Sex. Reprod. Health.

[35] VanBenschoten H (2022). Impact of the COVID-19 pandemic on access to and utilization of services for sexual and reproductive health: A scoping review. BMJ Glob. Health.

[36] Censo 2022|IBGE. https://www.ibge.gov.br/estatisticas/sociais/trabalho/22827-censo-demografico-2022.html

[37] Ben J, Cormack D, Harris R, Paradies Y (2017). Racism and health service utilisation: A systematic review and meta-analysis. PLoS ONE.

[38] Robertson, M. M. *et al.* Racial/ethnic disparities in exposure to COVID-19, susceptibility to COVID-19 and access to health care—findings from a U.S. national cohort. *medRxiv*10.1101/2022.01.11.22269101 (2022).

[39] Hamed S, Bradby H, Ahlberg BM, Thapar-Björkert S (2022). Racism in healthcare: a scoping review. BMC Public Health.

[40] Paixão M, Rossetto I, Montovanele F, Carvano L (2010). Relatório Anual das Desigualdades Raciais no Brasil: 2009–2010.

[41] Olinto MTA, Olinto BA (2000). Raça e desigualdade entre as mulheres: um exemplo no sul do Brasil. Cad. Saúde Pública.

